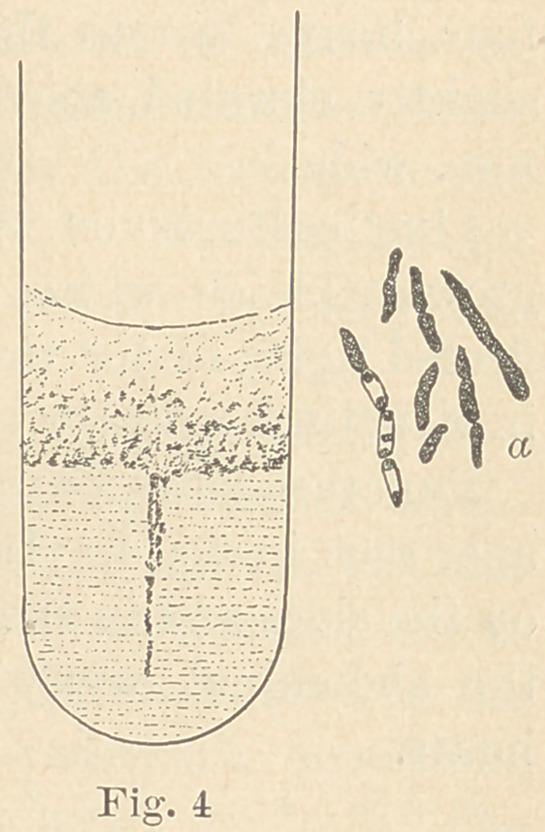# Pathogenic Bacteria of the Human Mouth

**Published:** 1888-07

**Authors:** W. D. Miller

**Affiliations:** Berlin, Germany


### Independent Practitioner.

Vol. IX. July, 1888. No. 7.

Note.—No paper published or to be published in another journal will be accepted for this department. All papers must be in the hands of the Editor before the first day of the month preceding that in which they are expected to appear. Extra copies will be furnished to each contributor of an accepted original article, and reprints, in pamphlet form, may be had at the cost of the paper, press-work and binding, if ordered when the manuscript is forwarded. The Editor and Publishers are not responsible for the opinions expressed by contributors. The journal is issued promptly, on the first day of each month.

### Original Communications.

### PATHOGENIC BACTERIA OF THE HUMAN MOUTH.

BY PROF. W. D. MILLER, BERLIN, GERMANY.

Continued From Page 284.

The first experiments upon animals showing "the poisonous nature of the human saliva" were, I believe, made in America. The injection of saliva under the skin of small animals was frequently seen to be followed by septicæmia and death of the animal in a few days, or even hours. Similar results were obtained by Pasteur, Raynaud and Lannelongue, in Paris.

A. Fränkel[1] mixed saliva from healthy persons with bouillon, and allowed the mixture to stand four to six hours at blood temperature. Rabbits vaccinated subcutaneously, or in the lung, died from blood poisoning in twenty-four to forty-eight hours. Occasionally Fränkel obtained the same results by vaccinating animals with fresh saliva. Also Miller,[2] by inoculating rabbits and mice with the saliva of an individual suffering from mycosis tonsillaris benigna. Inoculation in the lung was regularly followed by death of the animal within thirty hours. In like manner the experiments of Davaine,

[1] Deutsche Med. Wochenschrift, No. 25, 1882.
[2] Ibid.

Vulpian, Klein, Sternberg and others proved, beyond a doubt, that a group of micro-organisms is frequently to be met in the human mouth, which, having found their way into the circulation, may produce the most dangerous diseases.

Kreibohm[1] has furnished an important contribution to our knowledge of the pathogenic micro-organisms of the human mouth. He found, in the first place, two kinds of bacteria, which were characterized by the fact that they grew on none of the culture media now in use. The first kind was obtained twice by inoculating mice with the scrapings of a coated tongue. The mice died in a few days, and showed, on section, large numbers of bacilli in the blood. One drop of blood of these mice, inoculated into other mice, produced constantly the same disease. Death followed, as a rule, in two to three days.

The second kind was obtained in the same manner. Inoculation with this bacterium produced death in eighteen to forty hours. The micro-organisms were found in great numbers in the blood and in the capillaries of the different organs. They appeared as short rods, slightly contracted in the middle; after staining they have the appearance of an 8. Kreibohm also found once in the coating of the tongue and twice in saliva, a bacterium which he named Bacillus crassus sputigenus. It appeared as short, thick bacilli, with rounded ends, or often bent in the form of a sausage. The cultivation succeeded easily on different media. Mice die in about forty-eight hours after inoculation with small quantities, and show in the blood numerous bacilli. Rabbits do not react on slight vaccination, but die from blood poisoning forty-eight hours after intra-venous injections.

Black[2] examined the fluids in the mouth for pyogenic bacteria, and found in ten examinations, the Staphylococcus pyogenes aurens seven, Staph. pyog. albus four, and Streptococcus pyogenes three times. He came to the conclusion that a careful examination would reveal these micro-organisms in nearly all mouths.

The Micrococcus tetragenus has been repeatedly found by myself and others in the fluids of the mouth. It possesses, as is well known, pathogenic properties, and causes the death of small animals (mice, guinea-pigs, etc.) in three to ten days after vaccination.

---

[1] Flügge, Mikroorganismen, S. 257.

[2] INDEPENDENT PRACTITIONER, August, 1887.

Still more recently, Biondi[1] has described five pathogenic organisms obtained from the human mouth :—

1. Bacillus salivarius septicus.
2. Coccus salivarius septicus.
3. Micrococcus tetragenus.
4. Streptococcus septo-pyæmicus.
5. Staphylococcus salivarius pyogenes.

The Bacillus sal. sep. forms very short elliptic rods, with pointed ends and relatively thick body, and grows only slowly on the ordinary neutral media. Mice and rabbits, after injection of $\frac{1}{2}$-1 ccm. of saliva containing this micro-organism, died generally in twenty-four to seventy-two hours; the section showed œdem hæmorrhage tumor of the spleen, etc. The Coccus sal. sep. was found by Biondi only once, in the mouth of a patient suffering from puerperal septicæmia. Mice, guinea-pigs and rabbits, inoculated subcutaneously, died in four to six days with cocci in the blood and tissues. The Streptococcus septo-pyæmicus was not to be distinguished from that of erysipelas, phlegmon and puerperal metritis. Its action was also similar. The Staph. sal. pyog. was found only once, in the saliva of a person suffering from angina scarlatina. All animals infected with this micro-organism reacted by formation of abscess at the point of vaccination.

Two of these organisms, described by Biondi, Staphylococcus sal. pyog. and Coccus sal. sept., were found, each but once, in the mouth of persons suffering from severe infectious diseases, and can therefore hardly be considered as oral bacteria, any more than the tubercle bacillus, which may always be found in the mouths of consumptives.

Notwithstanding the great amount of work done upon the bacteria of the human mouth in the last few years, an immense amount remains to be done before we can be said to have arrived at anything like a thorough knowledge of the oral bacteria. Indeed, this field of work is so large that the longer one works at it the more bewildering it becomes, until at last one despairs of ever being able to make a thorough study of all the many different kinds of bacteria met with in the human mouth. Some three years ago I had already isolated, and in part described, some sixty different bacteria from the oral cavity; my work being then interrupted, I allowed

[1] Bresl. ärztl. Zeitschrift, Sept., 1887, No. 18.

nearly all of the cultures to die out. Among the fifty to sixty different kinds which I have cultivated in the last eighteen months, I recognize very few which I might possibly consider identical with any of those previously isolated. I have, consequently, from first to last, found more than one hundred species of bacteria in the human mouth. Any one who has done even a very little work in experimental bacteriology will at once recognize the absolute impossibility of any one person making a detailed study of one hundred different kinds of bacteria. One kind may furnish material for a life-work. I have, consequently, aimed only at general results, and in the case of but a very few have I attempted to make a more thorough study.

I have experimented with forty-two pure cultures, two mixed cultures, and twenty-two gangrenous pulps, and have made ninety-three subcutaneous inoculations of mice in pockets, using pure cultures, ten subcutaneous injections of pure cultures, fifty-eight pocket inoculations with pieces of gangrenous pulps, or with pus arising from such inoculations, sixty injections of pure culture into the abdominal cavity of mice, rabbits and guinea-pigs, twenty-two injections into the thoracic cavity, besides a number of mixed injections.

The pockets were made in the customary manner, at the root of the tail, and the material for inoculation was usually taken from an Agar-Agar culture one to two weeks old. Injections were made with the sterilizable subcutaneous syringe, cultures in beef-extract-peptone solutions from two to four days old, being used. For mice, 0.05 to 0.1 cc.; for rabbits and guineapigs, 0.25 to 0.5 cc., were injected. The mice were always etherized before making the injection. The etherization renders the operation much easier and surer; it may be accomplished in fifteen seconds by taking the mouse by the tail and poking him into a wide-mouthed ether bottle.

In 18.8 per cent. of the pocket inoculations a severe local reaction followed, resulting in the formation of a small abscess, generally remaining superficial, but occasionally penetrating into the subcutaneous tissue. In eight cases the inoculation was followed by death, the mice showing, in three cases, symptoms of blood poisoning, the micro-organisms being also present in the blood and different organs. In a number of cases necrosis of the skin around the pocket occurred, a piece of skin one-fourth to one-half inch in diameter being thrown off. In 50 per cent. the reaction was light, nothing more than a slight local redness and formation of a very minute quantity of pus being observed. In 31.2 per cent. no reaction whatever could be detected, the wound healing rapidly, without either suppuration or swelling. Of the different kinds tested by injections, 24 per cent. produced violent reactions, resulting either in the death of the animal from septicæmia, peritonitis, pleuritis, etc., or in extensive suppuration and abscess formation. Slight reaction was produced in 32 per cent., temporary sickness, from which the animals soon recovered, or slight swelling at the point of injection; in 44 per cent. no effect could be detected.

Subcutaneous inoculation with portions of gangrenous pulps produced comparatively severe symptoms in 36.8 per cent. of the pulps experimented with; slight effects in 47.4 per cent., and no apparent reaction in 15.8 per cent.

It appears from these results that inoculation with portions of gangrenous pulps is more dangerous than inoculation with pure cultures from the same pulps, which is as we should naturally expect it to be. I intend, however, later to discuss the question of the bacteria of foul pulps at length, and pass the subject here with this brief mention.

The mixed infections invariably resulted in the death of the animal.

During these studies I have found in the oral cavity a number of bacteria which possess more or less pathogenic action, four of which I have examined more in detail.

Fig. 1

The first of these, Micrococcus gingivæ pyogenes, was found in a case of pyorrhœa alveolaris three times in the same mouth, at intervals of three months; also in a very filthy mouth, in the deposit around the teeth. It appears as irregular cocci, or very plump rods, singly or in pairs. In gelatine-plate cultures it grows rapidly at room temperature, forming round colonies, with a distinctly sharp margin. At first the colonies appear very slightly colored under the microscope, and as they become older they grow very dark, especially where they lie far apart. Line cultures on Agar-Agar present a moderately thick, greyish growth, having a tinge of purple by transmitted light. Under the microscope they appear as a homogeneous, nearly colorless matrix, interspersed with darker figures of various irregular shapes.

Fig. 2

Puncture (*stich*) cultures in gelatine have, when eight days old, the appearance seen at *a*, fig. 1. The gelatine does not become liquefied. Cultures in beef-extract-peptone-sugar-solutions show a strong acid reaction and develop considerable quantities of gas. Subcutaneous inoculations of mice were followed by abscess and necrosis of the skin, occasionally resulting in the death of the animal. Injections in the abdominal cavity invariably produced the death of the animal in twelve to twenty-four hours, the section revealing immense numbers of bacteria in the abdominal cavity, a considerable quantity of a serous exudation, peritonitis, etc. Only a very limited number of larger animals—two rabbits and two guinea-pigs—have been inoculated. The animals appeared sick for a time, sitting quietly in the corner of the cage and refusing to eat. After two or three days, however, all symptoms disappeared.

Fig. 3

The second, Bacterium gingivæ pyogenes, was found in the same mouth with the micro-organisms just described, and also in a suppurating tooth-pulp. It appears in form of thick, short bacteria with rounded ends, one and a half to four times as long as thick, (see fig. 2, *a.*) In plate cultures it grows very rapidly, even at room temperature, the colonies being clearly visible to the naked eye in twenty-four hours. Under the microscope they appear as beautiful, perfectly round, yellowish colonies, with a sharp, dark border. The gelatine becomes rapidly liquefied, so that in forty-eight hours the first dilution is completely melted.

Line cultures on gelatine appear in fifteen hours as a trough of melted gelatine one and a half mm. broad, the side of the trough being cloudy and the bottom marked by a line of white sediment.

Line cultures in Agar-Agar present a thick, moist, slightly greyish growth by transmitted light, having a slight greenish yellow tinge under the microscope, colorless at the margin, yellowish brown towards the middle, and presenting a fibrillated structure.

Puncture cultures in gelatine, eight days old, have the appearance seen in fig. 2. The gelatine rapidly melts in form of a funnel, while the masses of bacteria sink to the bottom, the melted gelatine, however, remaining cloudy.

Injection of this fungus into the abdominal cavity of white mice produced death in ten to twenty-five hours. During their sickness the mice sit drawn up, with bent back and eyelids glued together. The section showed peritonitis, and in some cases purulent exudation. Micro-organisms were found only in very few numbers in the blood.

Injection of 0.25 into the abdominal cavity of rabbits and guinea-pigs produced identical results. Injection into the lung produced death in less than twenty-four hours. Subcutaneous inoculation (injections) of mice, resulted in extensive abscess formation.

The third bacterium, which I have named Bacillus dentalis viridans, was found in the superficial layers of carious dentine. It appears as slightly curved, pointed rods, single or in pairs (fig. 3, *a*). It grows well in plate cultures at room temperature; the colonies under the microscope are nearly colorless, having but a slight yellow tinge; they are perfectly round, with a sharp contour, and show, when they do not lie too close together, two or three concentric rings. This bacterium is characterized by the production of a beautiful opalescent green coloring matter, which it imparts to the gelatine; the cell itself is not colored.

Fig. 4

Line cultures on Agar-Agar produce a very thin growth, with irregular margins, bluish by transmitted light, greenish grey by reflected light, and colorless under the microscope.

Puncture cultures on gelatine, eight days old, present the form seen in fig. 3.

Subcutaneous applications from pure cultures of this bacterium produced severe local inflammation and suppuration, and in one case death by blood poisoning, the bacteria being found in large numbers in the blood and tissues.

Injections into the abdominal cavity of white mice and guinea-pigs, produced death in sixty per cent. of the cases, in twenty-two hours to six days, from peritonitis. Bacteria could not be found in the blood microscopically, but cultures made from the blood of the heart developed pure cultures of the bacterium injected.

The fourth micro-organism with pronounced pathogenic action, Bacillus pulpæ pyogenes, was found in a gangrenous tooth-pulp. It occurs as bacilli, often slightly curved and pointed, either singly, in pairs or in chains of four to eight (fig. 4, *a.*) It grows moderately well in gelatine-plate cultures, the colonies appearing large and round, dark yellowish brown, with distinct margin.

Line cultures on gelatine begin to melt in eighteen to twenty-four hours, up to that time appearing as greyish, shining lines, slightly elevated above the surface of the gelatine and about one mm. wide.

Line cultures on Agar-Agar produce a moderately extensive growth, bluish white, glistening by transmitted light, grey by reflected light; under the microscope, granular, sometimes fibrillar in structure, grey, or in older colonies, yellowish.

Puncture cultures in gelatine, eight days old, present the appearance seen in fig. 4. It melts the gelatine with about equal rapidity on the sides and in the middle of the tube. Injections of 0.05 into the abdominal cavity proved fatal to mice in eighteen to thirty hours.

(TO BE CONTINUED.)

## Figures and Tables

**Fig. 1 f1:**
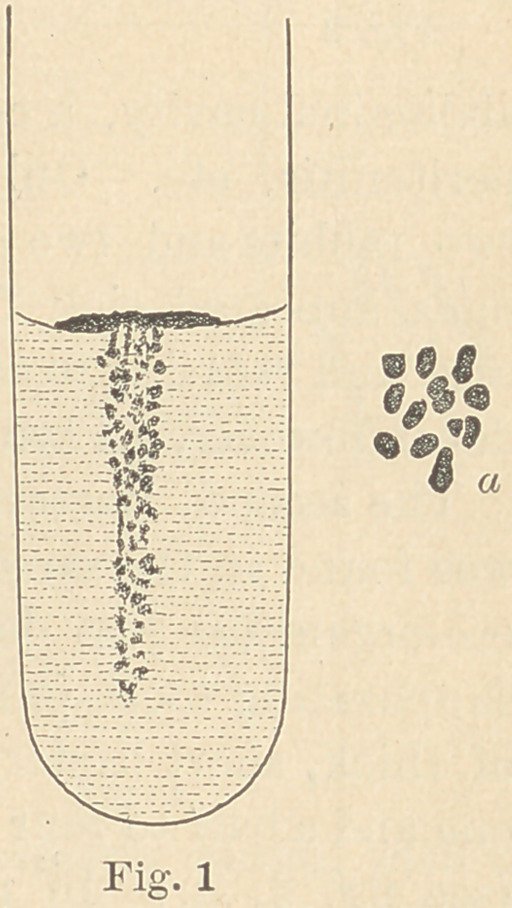


**Fig. 2 f2:**
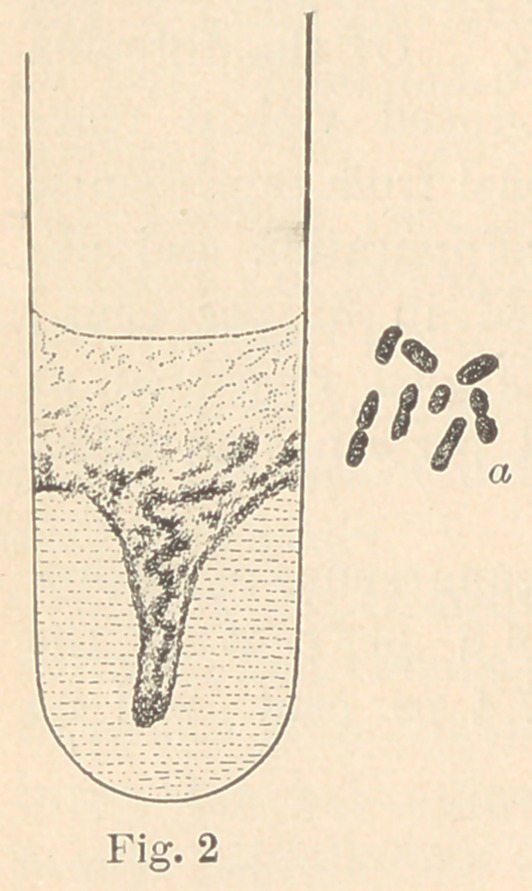


**Fig. 3 f3:**
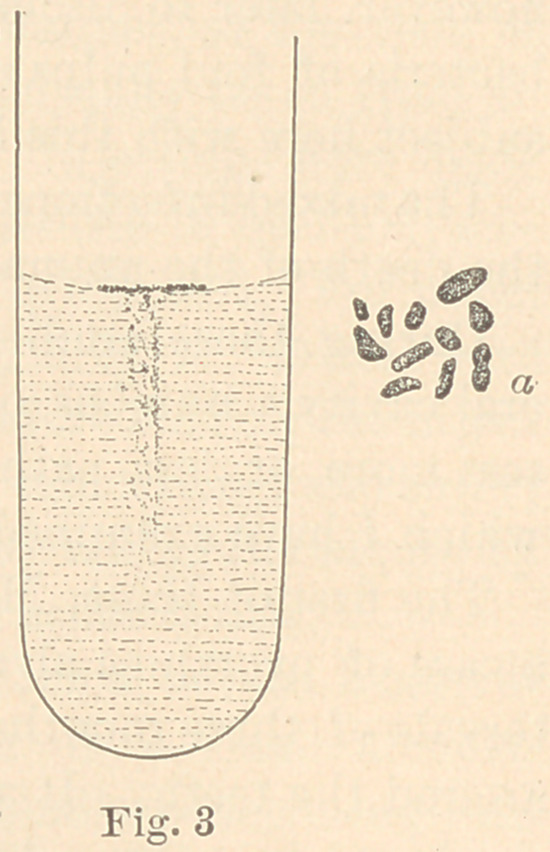


**Fig. 4 f4:**